# Cancer mortality in ethnic South Asian migrants in England and Wales (1993–2003): patterns in the overall population and in first and subsequent generations

**DOI:** 10.1038/sj.bjc.6605645

**Published:** 2010-04-27

**Authors:** P Mangtani, C Maringe, B Rachet, M P Coleman, I dos Santos Silva

**Affiliations:** 1 Department of Epidemiology and Population Health, London School of Hygiene and Tropical Medicine, Keppel Street, London WC1E 7HT, UK; 2 Cancer Research UK Cancer Survival Group, Department of Epidemiology and Population Health, London School of Hygiene and Tropical Medicine, Keppel Street, London WC1E 7HT, UK; 3 Cancer Research UK Epidemiology and Genetic Group, Department of Epidemiology and Population Health, London School of Hygiene and Tropical Medicine, Keppel Street, London WC1E 7HT, UK

**Keywords:** cancer mortality, migrants: England and Wales, Asian continental ancestry group, health transition

## Abstract

**Background:**

Cancer mortality has been examined among ethnic South Asian migrants in England and Wales, but not by generation of migration.

**Methods:**

Using South Asian mortality records, identified by a name-recognition algorithm, and census information, age-standardised rates among South Asians, and South Asian *vs* non-South Asian rate ratios, were calculated.

**Results and conclusions:**

All-cancer rates in ethnic South Asians were half of those in non-South Asians in first-generation (all-cancer-standardised mortality ratio (SMR) in males 0.51 and in females 0.56) and subsequent-generation South Asians (SMR in males 0.43 and in females 0.36). The higher mortality in first-generation South Asians for liver (both sexes), oral cavity and gallbladder cancer (females), particularly marked among Bangladeshis, was reduced in subsequent generations.

Landmark studies documenting changes in cancer risk in migrants from Japan to the United States, two populations with markedly different environmental exposures, highlighted their importance as causes of cancer (Buell and Dunn, 1965). South Asian ethnic migrants (with origins in the Indian subcontinent, irrespective of place of birth) are the largest group in England and Wales from low cancer incidence areas, comprising 4% of the population in 2001 (2.3 million) (Office for National Statistics, 2001). Peak migration into the United Kingdom of working-age South Asian adults occurred in the 1960s (Coleman and Salt, 1996). They have now reached age groups at which the incidence of most cancers is high, with offspring who are often adults, enabling an examination of cancer mortality patterns by generation. These patterns should provide clues to ethnic differentials in risk factors for cancer.

Early comparisons of cancer mortality between South Asians and native British populations were limited by the lack of population denominators and details the ethnicity of deceased persons (Balarajan *et al*, 1984; Marmot *et al*, 1984). Ethnic population denominators have been available since the 1991 census, but death certificates contain information only on the country of birth, a partial proxy for ethnicity: Caucasian British persons born in the Indian subcontinent during the colonial period up to 1947, as well as second-generation South Asians born in the United Kingdom, would be misclassified (Balarajan and Bulusu, 1990; Wild and Mckeigue, 1997; Wild *et al*, 2006).

We therefore took advantage of the fact that South Asians have distinctive names, allowing reliable separation from other ethnic groups in named data (Nicoll *et al*, 1986), such as mortality records. We present here the first national analyses of cancer mortality among South Asian ethnic migrants in England and Wales, including, for the first time, comparisons between first- *vs* subsequent-generation migrants. For the first generation, further analyses were conducted by country of birth.

## Methods

Mortality records for England and Wales were used from 1993 (when computerised mortality records with name information were available) to 2003. Of the 6 076 801 death records for these 11 years, we considered 1 526 139 (25%) records for analysis, in which the underlying cause of death was coded as neoplastic (ICD-9 codes 140–239 for 1993–2000; ICD-10 codes C00-D48 for 2001–03). Of these, 71 deaths were excluded because gender was missing or date of birth was later than date of death, and 16 946 deaths were coded as being due to a benign neoplasm or *in situ* malignancy (ICD-9 210-239, ICD-10 D00-D48, except for the central nervous system, for which benign tumours were retained) or non-melanoma skin cancer (ICD-9 173; ICD-10 C44). A total of 1 508 262 (98.8%) cancer deaths were included in the final analysis.

To ensure compatibility between the two ICD revisions, the number of deaths coded in ICD-9 (1993–2000) was multiplied by standard comparability ratios to give an ‘expected’ number of deaths if they had been coded using ICD-10. These ratios were calculated by the Office for National Statistics on the basis of bridge-coding exercises for mortality and are those that provide statistically significantly (*P*<0.05) different numbers of deaths between ICD revisions (Rooney *et al*, 2002; Brock *et al*, 2004). Age- and sex-specific comparability ratios were available for all-cancer mortality rates of the larynx (male), breast (women), prostate and colon–rectum (men and women), but only sex-specific (all ages) ratios for other cancers. Comparability ratios ranged from 1.010 for male oesophageal cancer to 1.061 for male leukaemia.

We used SANGRA (South Asian Names and Group Recognition Algorithm) to assign an ethnicity code to each death record. This computer algorithm identifies South Asian names by comparing names in a database with a dictionary of common South Asian names and spelling variants. It identified 14 650 potential South Asian names among 1 508 262 deaths at all ages.

SANGRA has a sensitivity of 91–96% and a specificity of 94–98% compared with self-assigned ethnicity (Nanchahal *et al*, 2001). Despite high specificity, it yields a low positive predictive value for South Asian ethnicity, given the low proportion of true South Asians in mortality records. To improve its predictive value, we ran a computerised query to exclude from the initial SANGRA positives records with (i) common English first names; (ii) non-South Asian first names or surnames compiled from previous visual inspections of algorithm positives (dos Santos Silva *et al*, 2003); and (iii) countries of birth that do not have large South Asian populations (e.g., Cyprus, Turkey, Iraq, Iran, Somalia). The remaining records were visually inspected, as in previous studies (Matheson *et al*, 1990; Swerdlow *et al*, 1995; Winter *et al*, 1999), confirming 10 767 (73%) as South Asian (0.71% of all deaths). A random sample of 100 records per calendar year was checked to assess sensitivity. In total, 2 out of 1100 (0.16%) records were found to have South Asian names not identified by SANGRA.

The 1991 and 2001 censuses provided estimates of the South Asian and non-South Asian populations of England and Wales. For inter-censal years, South Asian populations were estimated by 5-year age group and sex using linear interpolation. These estimates were then subtracted from the Office for National Statistics mid-year estimates of the overall population of England and Wales to derive population figures for non-South Asians. These censuses recorded South Asian ethnicity under slightly different, although comparable, categories (Rees and Butt, 2004).

The 5-year age-specific mortality rates for all cancers combined and for the 20 most common cancers were calculated. Mortality rates, age adjusted to the hypothetical world standard population (Ferlay *et al*, 2004), were calculated for all ages (available from authors) and for ages 0–74 years. The truncated range was used because the numbers of cancer deaths among South Asians aged 75 years and over were small; moreover, cancer diagnoses may have been less accurate and ethnic minorities may have been more likely to emigrate later in life (Harding, 2003). Country-of-birth analyses were restricted to the United Kingdom, India, Pakistan and Bangladesh. For more robust comparisons, as numbers were small, standardised mortality ratios (SMRs) were calculated as the ratio between the observed number of deaths and the number that would have been expected if they had experienced the same age- and sex-specific mortality rates as non-South Asians in England and Wales in 2001; approximate 95% confidence intervals (CIs) were calculated (Breslow and Day, 1980). A generalised linear model with Poisson error structure was used to formally test for trends. Analyses were conducted using STATA 10 software (Stata Corporation, 2006).

## Results

**Figure 1:**
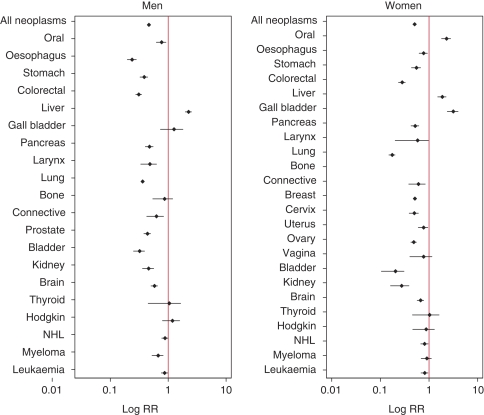
South Asian *vs* non-South Asian age-standardised mortality rate ratios for selected cancer sites by sex, in England and Wales, 1993–2003.

In ethnic South Asians, irrespective of the generation of migration, and in non-South Asians, the most common cancer deaths were from lung and prostate cancers in males, and from breast cancer in females (Table 1). Age-adjusted all-cancer mortality rates in South Asians were about half of those in non-South Asians (SMR and 95% CI in males 0.43 and 0.41–0.46, respectively, and in females 0.47 and 0.44–0.51, respectively) (Figure 1, Supplementary web table). Mortality rates in South Asians were significantly lower than those in non-South Asians for most cancers, except for liver cancers in both sexes, and oral cavity and gallbladder cancer in females.

Among non-South Asians, all-cancer mortality rates fell from 123 deaths per 100 000 in 1993–95 to 105 in 1999–2003, but there was little change among South Asians (52 per 100 000), producing a decrease in ethnic differentials over time (Supplementary web table). Mortality rates for non-South Asians decreased for stomach, colorectum and lung cancers in males, and for colorectum and breast cancer in females. Only lung cancer in males declined among South Asians, and more slowly than in their non-South Asian counterparts. The mortality advantage in South Asians was less marked, or even reversed, among younger adults for some cancers: stomach, colon and rectum combined, and leukaemia in both sexes; lung in males; and breast in females (Supplementary web figure). In contrast, for cervix cancer, the mortality advantage in South Asians was more marked for women under 45 years of age (0.16, 95% CI 0.08–0.24) than for older women (CI 0.62, 0.93). Analyses restricted to first-generation South Asian ethnic migrants produced slightly higher estimates of ethnic differentials than those based on country of birth, as the latter included both ethnic South Asian and non-South Asians (Table 2).

**Figure 2:**
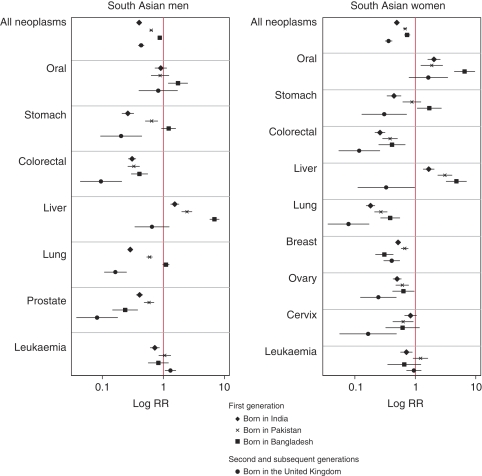
Cancer mortality (standardised mortality ratio, SMR) for selected sites in ethnic South Asians by sex in first-generation migrants born in India, Pakistan and Bangladesh, and in second and subsequent generations born in the United Kingdom, 1993–2003.

South Asian ethnic migrants of both sexes had lower all-cancer mortality rates than non-South Asians, regardless of the generation of migration (Figure 2, Table 2). First-generation South Asians born in India had the lowest all-cancer mortality rates relative to non-South Asians (SMR 0.40). The highest all-cancer SMRs among all first-generation South Asians were in men and women born in Bangladesh (SMR 0.88 and 0.73, respectively), reflecting their higher mortality from cancers of the lung, oral cavity, stomach, liver and gallbladder in both sexes, and from cancer of the oesophagus in women. Among first-generation South Asians, prostate and breast cancer mortality was highest among those born in Pakistan and lowest among those born in Bangladesh. The SMRs for all these cancers, apart from breast cancer, were lowest in second- or subsequent-generation South Asians as a whole, with liver cancer mortality being similar to that among non-South Asians.

A particular observation was made with leukaemia. Mortality was lower than in the general population for first-generation males born in India and Bangladesh, but not for those born in Pakistan or the United Kingdom (Table 2, Figure 2). Furthermore, the higher risk in ethnic South Asians born in Pakistan or the United Kingdom was restricted to those under 45 years of age (SMR in males and females was, respectively, 1.61 (CI 1.32–1.97) and 1.13 (CI 0.86–1.50) in those born in the United Kingdom, and 2.02 (CI 1.42–2.86) and 1.79 (CI 1.15–2.77) in those born in Pakistan). No such increased risk was seen in South Asians under 45 years of age born in India or Bangladesh.

## Discussion

Mortality from most cancers was lower in South Asians of first and subsequent generations than in non-South Asians, including the most common cancers among the general population of England and Wales. These low risks, first noted in the 1970s (Balarajan *et al*, 1984; Swerdlow *et al*, 1995), persist nearly 40 years later (Coleman and Salt, 1996), which is consistent with past incidence patterns (Winter *et al*, 1999; Rastogi *et al*, 2008; Jack *et al*, 2009; Metcalfe *et al*, 2009). Such low cancer rates could be seen as targets to aim for in the general population. For cancers related to infection (stomach and liver, although not cervical), higher mortality rates occurred in first-generation South Asians, especially among Bangladeshis, but this seems to have fallen in subsequent generations.

Sensitivity analyses to correct for potential under-ascertainment of ethnicity using SANGRA did not materially affect the estimates of trend in the rate ratios, although, as expected, it did slightly increase their mortality rates (data not presented). We used established procedures to reduce the number of false positives (non-South Asians incorrectly identified as South Asians); retaining them shifted the SMRs for South Asians closer to 1, as expected, but did not change the overall trends (data not presented). The category ethnic South Asian is broad, embracing differences in region, language and environment. Migration from Bangladesh is from poorer, more rural regions where fish and meat consumption is more common than in India, where lacto-vegetarianism is prevalent and educational levels may be higher (Mckeigue and Sevak, 1994; The Department of Health, 2001; Choudhury *et al*, 2007); this may explain some of the mortality difference in tobacco- and infection-related cancers between first-generation Bangladeshis, Indians and Pakistanis. The high mortality from tobacco-related cancers in Bangladeshi migrants indicates the need for better access to tobacco chewing and smoking cessation services (Zaman and Mangtani, 2007). The interesting pattern of higher prostate and breast cancer risks in migrants born in Pakistan, compared with India or Bangladesh, mirrors that seen in the countries of origin (Ferlay *et al*, 2004). Increased mortality among South Asians born in the United Kingdom from cancers associated with a Western lifestyle has not yet been seen, but numbers are small and these generations are still young, especially those with family roots in Bangladesh.

## Figures and Tables

**Table 1:** Cancer sites ranked by percentage of total South Asian (similarly non-South Asian) cancer deaths, in England and Wales, 1999–2003

			**Percentage of cases**		
**South Asian rank**	**ICD-10 codes**	**Site**	**South Asian**	**Non-South Asian**	**Non-South Asian rank**
*Men*					
1	C33, C34	Lung	19.8	25.2	1
2	C61	Prostate	9.2	12.7	2
3	C18-C21	Colorectal	7.0	10.7	3
4	C91-C95 except C91.4, C94.4, C94.5	Leukaemia	7.0	3.0	9
5	C22	Liver	7.0	1.7	11
6	C82-C85, C88.3, C91.4, C96.0-C96.3, C96.7, C96.9	NHL	6.5	3.0	8
7	C25	Pancreas	4.9	4.2	7
8	C70, C71, C72, D32, D33, D42, D43	Brain	4.8	2.4	10
9	C16	Stomach	4.1	5.1	5
10	C15	Oesophagus	3.1	5.6	4
11	C01-06, C09, C10, C12-C14	Oral	2.8	1.2	13
12	C67	Bladder	2.2	4.2	6
13	C90.0, C90.1, C88.7, C88.9	Myeloma	2.1	1.6	12
14	C32	Larynx	1.1	0.8	15
15	C64-C66	Kidney	1.1	1.0	14
16	C81	Hodgkin's disease	0.8	0.2	18
17	C47, C49	Connective tissue	0.7	0.4	16
18	C40, C41	Bone	0.7	0.2	19
19	C23, C24	Gall bladder	0.5	0.3	17
20	C73	Thyroid	0.3	0.1	20
					
*Women*					
1	C50	Breast	21.3	17.7	1
2	C56, C57.0-C57.4	Ovary	6.8	6.1	4
3	C33, C34	Lung	6.1	17.4	2
4	C82-C85, C88.3, C91.4, C96.0-C96.3, C96.7, C96.9	NHL	5.1	3.0	8
5	C18-C21	Colorectal	5.0	10.5	3
6	C91-C95 except C91.4, C94.4, C94.5	Leukaemia	4.5	2.6	9
7	C25	Pancreas	4.2	4.8	5
8	C22	Liver	4.0	1.3	15
9	C70, C71, C72, D32, D33, D42, D43	Brain	3.9	1.8	12
10	C15	Oesophagus	3.6	3.5	6
11	C01-06, C09, C10, C12-C14	Oral	3.2	0.7	16
12	C53	Cervix	2.9	1.6	14
13	C54, C55	Uterus	2.8	2.1	11
14	C16	Stomach	2.5	3.3	7
15	C90.0, C90.1, C88.7, C88.9	Myeloma	2.4	1.7	13
16	C23, C24	Gall bladder	2.2	0.5	18
17	C47, C49	Connective	0.8	0.5	19
18	C73	Thyroid	0.7	0.3	20
19	C67	Bladder	0.7	2.3	10
20	C51, C52	Vagina	0.6	0.7	17

Abbreviations: ICD-10=International Classification of Diseases–10th Revision, NHL=non-Hodgkin's lymphoma.

Note: On average between 1999 and 2003, unspecified cancers (ICD-10 C76-C80) account for 0.71% of non-South Asian cancer registrations and 0.54% of South Asians.

**Table 2:** Cancer mortality by country of birth for all ethnic groups and South Asians only, by sex and selected cancer sites, in England and Wales, 1993–2003^a^: no. of deaths, standardised mortality ratios^b^ (0–74 years)

					**South Asian ethnic group**																			
	**All ethnic groups born in**				**First generation**																**Second generation**			
**Country of birth**	**India, Pakistan or Bangladesh**				**India, Pakistan or Bangladesh**				**India**				**Pakistan**				**Bangladesh**				**United Kingdom**			
**Malignancy**	**Deaths**	**SMR**	**95% CI**		**Deaths**	**SMR**	**95% CI**		**Deaths**	**SMR**	**95% CI**		**Deaths**	**SMR**	**95% CI**		**Deaths**	**SMR**	**95% CI**		**Deaths**	**SMR**	**95% CI**	
*Men*																								
All neoplasms	7632	0.62	0.60	0.63	4793	0.51	0.49	0.52	2475	0.40	0.39	0.42	1484	0.63	0.60	0.67	835	0.88	0.82	0.94	332	0.43	0.39	0.48
Oral	210	0.95	0.83	1.09	146	0.85	0.72	0.99	82	0.91	0.73	1.13	35	0.88	0.64	1.23	29	1.74	1.21	2.50	7	0.83	0.40	1.71
Oesophagus	303	0.41	0.37	0.46	158	0.28	0.24	0.32	103	0.28	0.23	0.34	32	0.22	0.16	0.32	23	0.39	0.26	0.58	4	0.13	0.05	0.33
Stomach	345	0.57	0.51	0.63	207	0.45	0.39	0.51	80	0.26	0.21	0.33	72	0.64	0.51	0.81	55	1.22	0.94	1.59	6	0.20	0.09	0.44
Colorectal	626	0.48	0.44	0.51	327	0.33	0.29	0.36	204	0.31	0.27	0.35	82	0.33	0.26	0.41	41	0.41	0.30	0.55	6	0.10	0.04	0.21
Liver	488	1.91	1.75	2.09	400	2.04	1.85	2.25	169	1.54	1.33	1.79	107	2.43	2.01	2.94	124	6.90	5.79	8.22	9	0.65	0.34	1.25
Gall bladder	35	1.17	0.84	1.63	29	1.28	0.89	1.84	10	0.66	0.36	1.22	9	1.67	0.88	3.17	10	4.66	2.53	8.57	0	0.00	0.00	0.00
Pancreas	380	0.70	0.63	0.77	228	0.54	0.48	0.62	131	0.48	0.41	0.58	65	0.62	0.48	0.78	32	0.73	0.52	1.03	4	0.17	0.07	0.44
Larynx	99	0.87	0.72	1.06	64	0.73	0.57	0.94	41	0.74	0.54	1.00	14	0.62	0.37	1.05	9	0.98	0.52	1.85	1	0.22	0.04	1.23
Lung	1700	0.53	0.51	0.56	1085	0.45	0.42	0.47	458	0.29	0.26	0.32	355	0.60	0.54	0.66	272	1.10	0.98	1.24	22	0.16	0.11	0.25
Connective tissue	52	0.84	0.64	1.10	35	0.71	0.51	0.98	19	0.65	0.42	1.02	11	0.76	0.42	1.36	5	0.80	0.34	1.87	11	0.73	0.41	1.30
Prostate	785	0.62	0.58	0.67	399	0.43	0.39	0.48	264	0.41	0.36	0.46	118	0.58	0.49	0.70	17	0.24	0.15	0.38	6	0.08	0.04	0.18
Bladder	208	0.46	0.40	0.53	119	0.35	0.29	0.42	73	0.31	0.31	0.25	33	0.42	0.42	0.30	13	0.43	0.25	0.74	2	0.08	0.08	0.02
Kidney	194	0.57	0.50	0.66	126	0.48	0.40	0.57	65	0.95	0.95	0.75	47	1.69	1.69	1.27	14	1.20	0.72	2.02	5	0.81	0.35	1.91
Brain	212	0.57	0.50	0.65	155	0.52	0.44	0.61	82	0.38	0.38	0.30	55	0.53	0.53	0.41	18	0.41	0.26	0.65	53	0.77	0.59	1.01
Thyroid	18	1.01	0.64	1.60	15	1.09	0.66	1.80	7	0.80	0.80	0.39	6	1.71	1.71	0.78	2	1.42	0.39	5.18	0	0.00	0.00	0.00
Hodgkin's disease	45	1.46	1.09	1.96	34	1.36	0.97	1.90	17	1.22	0.76	1.96	12	1.56	0.89	2.73	5	1.49	0.64	3.49	9	1.23	1.23	0.65
NHL	415	1.02	0.93	1.12	309	0.98	0.87	1.09	155	0.78	0.78	0.67	130	1.56	1.56	1.32	24	0.69	0.47	1.03	29	0.84	0.58	1.20
Myeloma	168	0.83	0.72	0.97	112	0.73	0.61	0.88	62	0.63	0.63	0.49	39	1.05	1.05	0.77	10	0.69	0.38	1.26	1	0.12	0.02	0.64
Leukaemia	328	0.89	0.79	0.99	234	0.82	0.72	0.93	129	0.73	0.61	0.86	79	1.06	0.85	1.32	26	0.83	0.57	1.21	96	1.31	1.07	1.60
																								
*Women*																								
All neoplasms	6266	0.71	0.69	0.72	3579	0.56	0.54	0.57	2212	0.50	0.48	0.52	1022	0.68	0.64	0.72	345	0.73	0.66	0.82	232	0.36	0.32	0.41
Oral	160	1.92	1.64	2.24	118	1.90	1.59	2.28	70	2.04	1.61	2.58	23	1.87	1.24	2.82	25	6.55	4.45	9.65	7	1.64	0.79	3.43
Oesophagus	206	0.76	0.66	0.87	135	0.72	0.61	0.85	92	0.68	0.55	0.83	13	0.32	0.19	0.55	30	2.64	1.85	3.77	1	0.06	0.01	0.36
Stomach	179	0.72	0.62	0.83	106	0.62	0.51	0.74	55	0.44	0.34	0.58	33	0.88	0.62	1.23	18	1.71	1.08	2.70	5	0.31	0.13	0.72
Colorectal	450	0.56	0.51	0.61	171	0.30	0.26	0.35	107	0.26	0.22	0.32	49	0.38	0.29	0.51	15	0.41	0.25	0.68	6	0.12	0.05	0.26
Liver	210	1.62	1.41	1.85	172	1.87	1.61	2.17	92	1.67	1.36	2.05	54	3.08	2.37	4.02	26	4.78	3.25	7.03	3	0.33	0.11	0.97
Gall bladder	132	3.20	2.70	3.79	110	3.80	3.15	4.58	68	3.27	2.58	4.15	27	4.26	2.93	6.20	15	8.25	5.00	13.61	0	0.00	0.00	0.00
Pancreas	306	0.77	0.69	0.86	162	0.58	0.49	0.67	88	0.44	0.35	0.54	59	0.96	0.74	1.24	15	0.84	0.51	1.39	4	0.18	0.07	0.47
Larynx	16	0.78	0.48	1.27	13	0.89	0.52	1.51	7	0.68	0.33	1.39	3	0.90	0.31	2.66	3	2.99	1.02	8.80	0	0.00	0.00	0.00
Lung	647	0.41	0.38	0.44	243	0.21	0.19	0.24	147	0.18	0.15	0.21	68	0.27	0.21	0.34	29	0.38	0.27	0.55	6	0.08	0.04	0.17
Connective tissue	37	0.74	0.54	1.02	28	0.72	0.50	1.04	16	0.65	0.40	1.06	9	0.86	0.45	1.63	3	0.76	0.26	2.23	5	0.46	0.20	1.08
Breast	1263	0.72	0.68	0.76	718	0.54	0.50	0.58	460	0.52	0.48	0.57	224	0.67	0.59	0.76	34	0.31	0.22	0.43	42	0.41	0.30	0.55
Uterus	157	0.88	0.75	1.02	89	0.70	0.57	0.86	63	0.69	0.54	0.89	23	0.82	0.55	1.22	3	0.36	0.12	1.05	1	0.11	0.02	0.60
Cervix	141	0.75	0.64	0.89	112	0.76	0.63	0.91	77	0.83	0.67	1.04	26	0.63	0.43	0.92	9	0.62	0.32	1.17	3	0.17	0.06	0.49
Ovary	425	0.68	0.62	0.75	250	0.54	0.47	0.61	159	0.50	0.43	0.59	68	0.61	0.48	0.77	23	0.64	0.42	0.96	8	0.25	0.12	0.49
Bladder	89	0.55	0.45	0.68	27	0.25	0.17	0.36	18	0.22	0.14	0.35	7	0.31	0.15	0.63	2	0.33	0.09	1.21	2	0.19	0.05	0.68
Kidney	98	0.65	0.53	0.79	46	0.43	0.32	0.57	29	0.93	0.65	1.34	14	1.38	0.82	2.31	3	0.97	0.33	2.83	8	2.08	1.06	4.10
Brain	134	0.62	0.53	0.74	102	0.61	0.50	0.74	63	0.44	0.34	0.56	30	0.52	0.37	0.75	9	0.44	0.23	0.84	20	0.36	0.23	0.55
Thyroid	36	1.58	1.14	2.18	23	1.44	0.96	2.16	14	1.24	0.74	2.08	6	1.71	0.78	3.73	3	2.91	0.99	8.55	0	0.00	0.00	0.00
Hodgkin's disease	20	0.97	0.63	1.50	13	0.77	0.45	1.33	7	0.76	0.38	1.55	5	1.09	0.48	2.50	0	0.00	0.00	0.00	7	1.48	0.73	3.01
NHL	282	1.08	0.96	1.22	175	0.93	0.80	1.07	109	0.83	0.69	1.01	58	1.31	1.01	1.70	8	0.57	0.29	1.13	14	0.62	0.37	1.03
Myeloma	143	1.04	0.88	1.22	94	0.98	0.80	1.19	57	0.82	0.63	1.06	30	1.46	1.02	2.07	7	1.21	0.59	2.48	1	0.14	0.03	0.77
Leukaemia	206	0.91	0.79	1.04	138	0.83	0.70	0.98	79	0.71	0.57	0.89	50	1.22	0.92	1.61	9	0.66	0.35	1.24	51	0.95	0.72	1.25

Abbreviations: CI=confidence interval; NHL=non-Hodgkin's lymphoma; SMR=standardised mortality ratio.

a SMRs for the periods 1993–96, 1997–2000 and 2001–03 are presented in the Supplementary web table.

b Observed number of deaths in under 75 year olds only. Expected number of deaths based on cancer specific/all neoplasm death rates in non-South Asians in 2001 in England and Wales.
